# Sudden Cardiopulmonary Arrest in a Neonate Eight Days After Being Infected With SARS-CoV-2

**DOI:** 10.7759/cureus.86393

**Published:** 2025-06-19

**Authors:** Takuya Wakamiya, Hideaki Ueda, Mio Tanaka

**Affiliations:** 1 Cardiology, Kanagawa Children's Medical Center, Yokohama, JPN; 2 Pathology and Diagnostics, Kanagawa Children's Medical Center, Yokohama, JPN

**Keywords:** cardiopulmonary arrest, covid-19 infection, hemorrhage, pediatric patient, thromb

## Abstract

COVID-19 infection typically presents with mild symptoms in pediatric patients; however, rare but serious complications can occur, including fatal outcomes. This case report describes the clinical course and autopsy findings of a one-month-old female infant who initially presented with fever and bloody stools and subsequently developed severe coagulopathy and hemorrhage associated with COVID-19. The infant experienced cardiopulmonary arrest and succumbed to irreversible global brain dysfunction despite intensive care. Autopsy revealed significant intramyocardial hemorrhage and fibrinoid degeneration in the coronary arteries and intestinal microvasculature, underscoring the potential for severe vascular complications in infants with COVID-19. These findings highlight the importance of considering systemic involvement and potential coagulopathies in the management of pediatric COVID-19 cases.

## Introduction

The clinical manifestations of COVID-19 infection are diverse, with the most commonly reported symptoms being fever, cough, and dyspnea. However, the symptomatology extends beyond the respiratory system to include a variety of other symptoms such as myalgia, headache, gastrointestinal disturbances, and anosmia. While the course of COVID-19 infection in pediatric patients is generally benign, it is important to recognize that serious and even fatal outcomes can occur.

Epidemiologic studies have provided valuable insight into the morbidity and mortality associated with COVID-19 in pediatric patients [1]. For example, a 2022 Japanese study documented 62 deaths in patients under the age of 20, highlighting that serious complications are a reality in this age group. A detailed analysis of these cases revealed that 53 had a known cause of death, with 46 attributed to internal factors and seven to external causes. Among the internal causes, central nervous system abnormalities were the most common, followed by circulatory and respiratory abnormalities, other known causes, and a subset of unexplained cases [1]. These data underscore the importance of a comprehensive evaluation of pediatric COVID-19 patients, even if initial symptoms appear mild.

## Case presentation

The patient was a one-month-old female infant who was initially admitted to a regional general hospital due to the onset of fever and the presence of bloody stools. A rapid COVID-19 test was positive. Of note, the infant had no respiratory symptoms on admission and was in good general condition. However, because of her young age, it was decided to admit her to the hospital for close observation.

Over the next few days, the infant's clinical course evolved. By the third day of hospitalization, the fever had resolved, and her general condition appeared stable. Despite the improvement in fever, the bloody stools persisted, raising concerns about possible underlying etiologies. In response to this persistent symptom, a tentative diagnosis of milk allergy was considered, and the infant was placed on a fasting control as part of the diagnostic and therapeutic approach.

On the morning of the fifth day of hospitalization in a regional general hospital, a sudden and critical event occurred: the infant began crying and abruptly went into cardiopulmonary arrest. The medical team immediately began cardiopulmonary resuscitation, and after about an hour, spontaneous circulation was successfully restored. However, resuscitation efforts revealed a significant cardiac conduction abnormality: the infant was bradycardic with third-degree atrioventricular block, with a heart rate of approximately 60 beats per minute.

Given the complexity of the infant's condition and the need for specialized care, she was transferred to our hospital while receiving intermittent cardiopulmonary resuscitation to maintain circulatory support. Upon arrival at our institution, a comprehensive evaluation was performed. Echocardiography revealed a severely reduced ejection fraction of only 30%, indicating severely impaired cardiac function. This, combined with the persistent bradycardia, resulted in profound hypotension. Attempts to obtain a measurable blood pressure were initially unsuccessful.

Percutaneous pacing was attempted to control the bradycardia; however, this intervention proved ineffective. Recognizing the severity of the situation, the medical team initiated extracorporeal membrane oxygenation (ECMO) to provide temporary support for the patient's cardiac and respiratory functions. Upon initiation of ECMO, a positive response was observed: atrioventricular block improved, heart rate increased to 120 beats per minute, and blood pressure became measurable at 70 mmHg, leading to hemodynamic stabilization.

Despite the initial stabilization achieved with ECMO, further evaluation revealed devastating neurological injury. On the second day after transfer to our hospital, electroencephalography (EEG) showed almost flat activity, indicating severe brain dysfunction. Clinical evaluation revealed the absence of brainstem reflexes, leading to the diagnosis of irreversible global brain dysfunction with no possibility of neurological recovery. A repeat EEG performed on the third day confirmed the initial findings.

Subsequently, the infant's overall condition continued to deteriorate, affecting both respiratory and hemodynamic parameters. Given the irreversible neurological injury and progressive deterioration of other organ systems, a multidisciplinary team, including physicians and the infant's parents, engaged in extensive discussions regarding the most appropriate course of care. Ultimately, the decision was made to transition to palliative care with a focus on comfort and support. The infant died on the third day after transfer to our facility.

**Figure 1:**
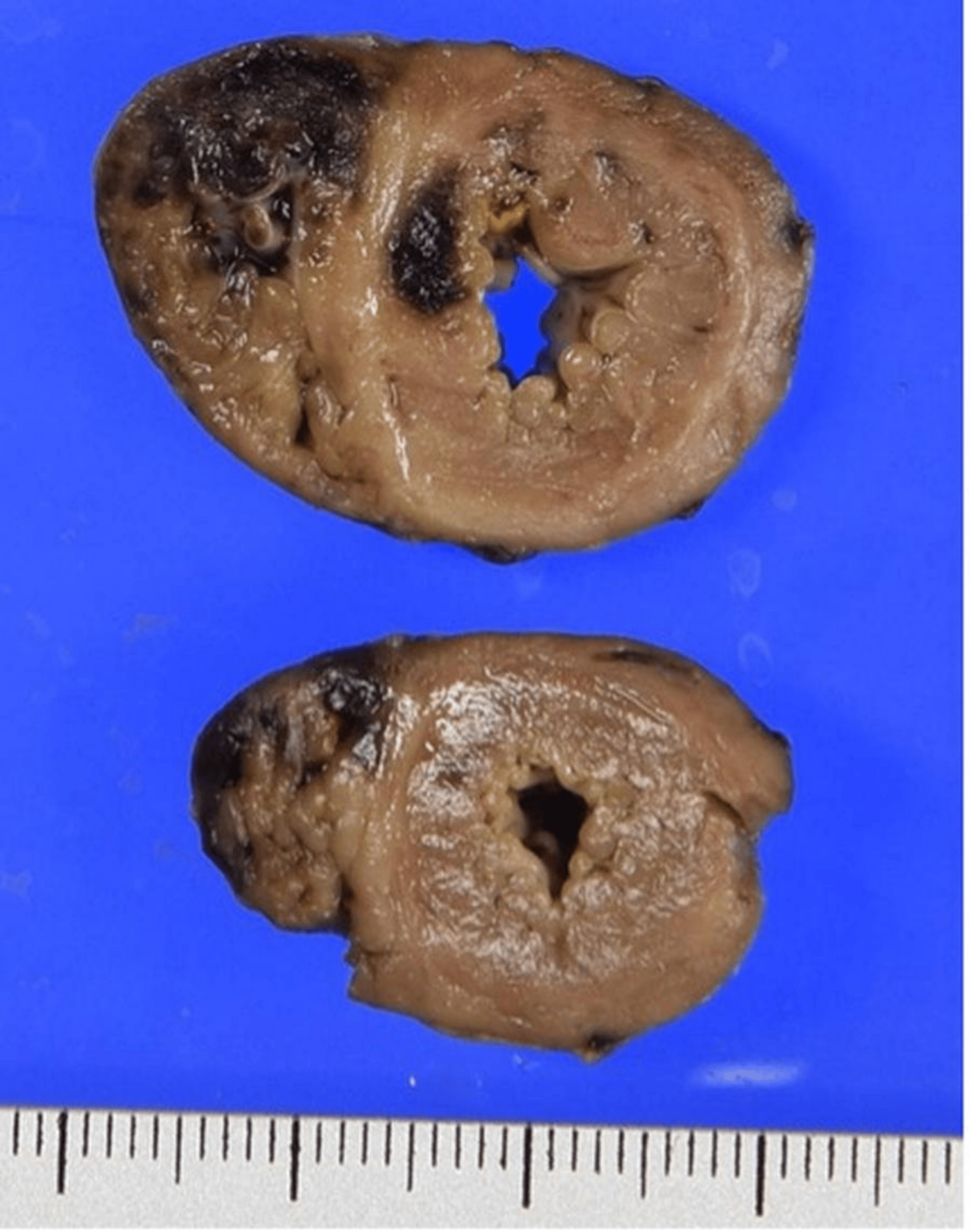
Cross-sections of the heart showing dark reddish hemorrhage and yellowish necrosis, mainly in the right ventricle and ventricular septum

**Figure 2:**
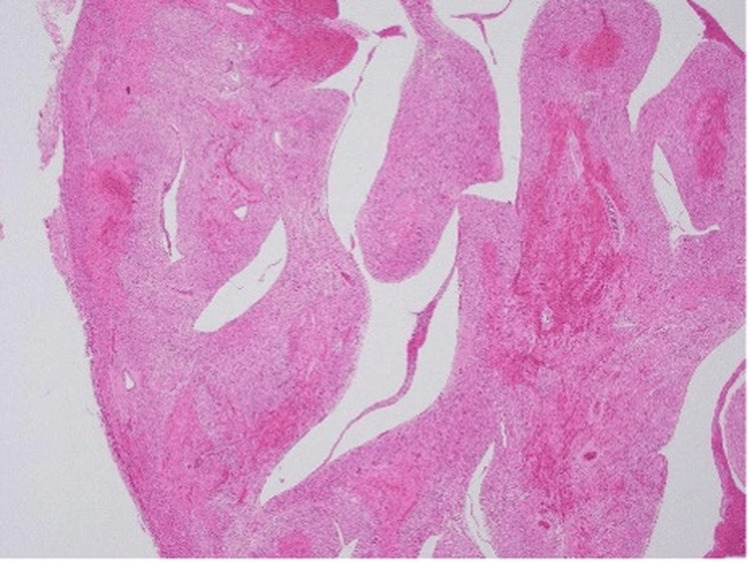
Irregularly shaped hemorrhagic and necrotic foci confirmed throughout the epicardium to endocardium of the myocardial wall (hematoxylin and eosin stain, original magnification ×40)

**Figure 3:**
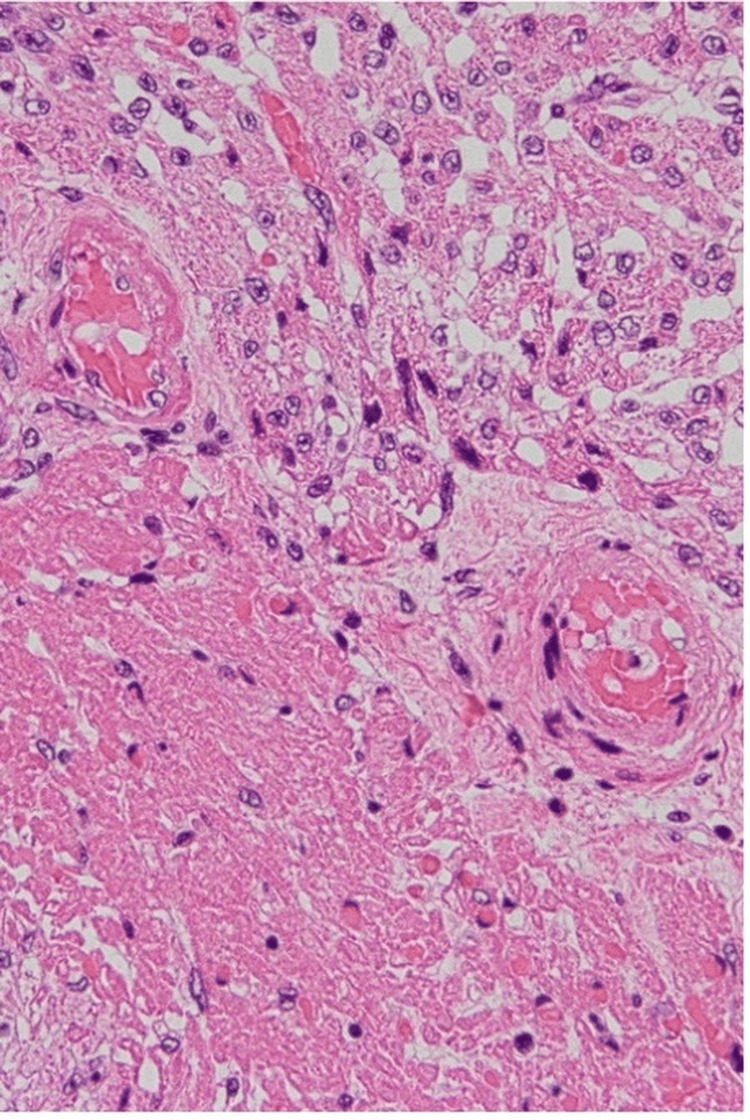
Fibrinoid degeneration in small vessels observed in the areas of hemorrhagic necrosis of the myocardium (hematoxylin and eosin stain, original magnification ×200)

To gain a deeper understanding of the pathological processes involved, the parents consented to an autopsy. The autopsy findings provided critical insight into the pathophysiological mechanisms underlying the infant's clinical deterioration and eventual death. The examination revealed significant intramyocardial hemorrhage in both the left and right ventricles and the ventricular septum (Figure 1). In addition, fibrinoid degeneration and hemorrhage were observed in the coronary arteries, indicating significant vascular damage within the heart (Figures 2-3).

Interestingly, despite the extensive myocardial damage, there were only sparse inflammatory infiltrates in the myocardium. This observation suggested that myocarditis, a common cause of cardiac dysfunction, was unlikely to be the primary pathological process in this case. Instead, the findings suggested a primary vascular etiology.

Further examination revealed fibrinoid necrosis of the arterioles in the submucosa of the small intestine and the subserosa of the gallbladder. This finding explained the bloody stools observed during the infant's initial presentation and subsequent hospitalization. Hemorrhage was also seen in a number of other organs, including the spleen, thymus, pancreas, gallbladder, and bladder, suggesting a systemic hemorrhagic disorder. However, other than the hemorrhagic findings, there was no significant evidence of inflammation in the organs examined.

## Discussion

The pathology findings strongly suggested that the cause of death was coagulopathy and hemorrhage secondary to COVID-19 infection. This conclusion highlights the potential for severe and life-threatening vascular complications in infants with COVID-19, even in the absence of prominent respiratory symptoms. This feature distinguishes neonatal cases from the typical adult presentation. Among the pathological features reported in COVID-19-related cardiac injury, macrophage infiltration is the most frequent (observed in 86% of cases), while microvascular and endocardial thrombi are less commonly noted, at 19% and 14%, respectively [2]. Another multicenter autopsy review documented microvascular thrombi in a median of 36.2% of cases [3], confirming the vascular injury as a critical component of myocardial pathology in COVID-19. Two primary mechanisms have been proposed to explain the pathogenesis of COVID-19-related thrombosis. First, SARS-CoV-2 may directly infect cardiomyocytes and vascular endothelial cells via the ACE2 receptor, leading to cytotoxic injury and endothelial dysfunction, thereby promoting a prothrombotic state [4]. Second, the host immune response, particularly through a cytokine-mediated hyperinflammatory process, can result in microvascular damage and a systemic procoagulant state known as immunothrombosis. This so-called cytokine storm disrupts normal hemostatic balance and contributes to both thrombosis and secondary hemorrhage [5].

In the present case, autopsy revealed fibrinoid degeneration and hemorrhagic necrosis within the myocardial microvasculature. This finding supports the hypothesis that microvascular injury, rather than classical myocarditis, was the primary driver of cardiac dysfunction, ultimately leading to conduction disturbance, bradycardia, and cardiac arrest. These findings are consistent with a growing body of pediatric and adult autopsy reports indicating that COVID-19-associated myocardial injury may often be ischemic and vascular rather than inflammatory in origin [2,3]. Furthermore, the gastrointestinal bleeding initially attributed to milk allergy was later found to be a consequence of microvascular fibrinoid degeneration in the intestinal submucosa. This underscores the systemic nature of the coagulopathy in this infant and illustrates how initial symptoms can be misleading. This case shares overlapping features with multisystem inflammatory syndrome in children (MIS-C), a hyperinflammatory response observed in pediatric populations following COVID-19. MIS-C is characterized by fever, shock, and multi-organ involvement, often including the heart. However, our patient did not fulfill the full criteria for MIS-C. The presence of gastrointestinal hemorrhage, myocardial dysfunction, and systemic coagulopathy places this case within the broader spectrum of pediatric COVID-19-associated inflammatory responses [6,7].

From a clinical standpoint, the management of thrombotic complications in pediatric COVID-19 cases remains underdefined. In adults, the use of anticoagulation (e.g., low-molecular-weight heparin) is widely recommended for high-risk patients [6]. Given increasing reports of coagulopathy and thrombotic events in children, especially in those with MIS-C or severe systemic illness, it is reasonable to consider thromboprophylaxis in select pediatric patients, particularly neonates and infants with persistent gastrointestinal bleeding, cardiovascular instability, or elevated D-dimer levels [8]. Ultimately, this case highlights the importance of maintaining a high level of clinical suspicion and conducting comprehensive monitoring in infants with COVID-19. Early recognition of systemic signs beyond the respiratory system, such as unexplained bleeding, bradycardia, or altered level of consciousness, should prompt a rapid evaluation of cardiac and coagulation function, even in the absence of respiratory symptoms.

## Conclusions

This case report highlights the potential for serious and life-threatening complications of COVID-19 infection in infants. The case presented highlights the occurrence of fatal coagulopathy and hemorrhage in a one-month-old infant, with autopsy findings of extensive microvascular damage. These findings underscore the importance of considering systemic involvement and the possibility of coagulopathies in the evaluation and management of pediatric COVID-19 cases. Furthermore, this case supports the consideration of thromboprophylaxis in pediatric patients with COVID-19, especially in the presence of risk factors or evidence of vascular or coagulation abnormalities. Further research is warranted to better understand the pathogenesis of COVID-19-related vascular complications in pediatric patients and to develop evidence-based guidelines for the prevention and treatment of these complications.
